# Supplementary material for: Control of working memory by phase–amplitude coupling of human hippocampal neurons
Source: Nature. 2024 Apr 17;629(8011):393–401. doi: 10.1038/s41586-024-07309-z (PMC11078732; doi:10.1038/s41586-024-07309-z)
Supplement: Supplementary file 1 — Supplementary Tables 1–5, Supplementary Discussion and Supplementary References. [file 41586_2024_7309_MOESM1_ESM.pdf]

nature portfolio

https://doi.org/10.1038/s41586-024-07309-z

Supplementary information

Control of working memory by phase–amplitude coupling of human hippocampal neurons

In the format provided by the authors and unedited

Supplementary tables

Area	Slow theta - low gamma	Slow theta - high gamma	Fast theta - low gamma	Fast theta - high gamma
Hippocampus	t(149) = -1.69, p = 0.0973	t(147) = -2.3141, p = 0.0163	t(121) = -0.14, p = 0.8956	t(116) = -3.05, p = 0.0015
Amygdala	t(162) = 0.37, p = 0.7104	t(138) = 0.54, p = 0.5896	t(131) = -0.63, p = 0.5252	t(93) = 0.96, p = 0.3431
pre-SMA	t(17) = 1.12, p = 0.2845	t(26) = -0.92, p = 0.3629	t(4) = -0.22, p = 0.8676	0 significant channels selected
dACC	t(33) = 0.46, p = 0.6644	t(12) = -1.23, p = 0.2385	t(19) = -0.65, p = 0.5276	t(8) = 1.78, p = 0.1203
vmPFC	t(64) = 1.37, p = 0.1771	t(48) = 0.01, p = 0.9916	t(29) = 0.36, p = 0.7200	t(27) = -0.55, p = 0.5774

Table S1. PAC comparisons between the load conditions for each combination of slow (2-5 Hz) and fast theta (5-9 Hz) with low (30-55 Hz) and high gamma (70-140 Hz) in each area. Two-sided permutation-based t-tests with no corrections for multiple comparisons. Related to Figure 2.

RT ~ 1 + PAC + load + PAC * load + (1 | patientID) + (1 | patientID:channelID)

Hippocampus

Name	Estimate	t	p
**Intercept**	0.38	7.76	9×10^{-15}
**PAC**	-49.07	-4.01	6×10^{-5}
**Load1**	-0.48	-7.10	1×10^{-12}
**PAC : Load1**	26.30	1.58	0.11

Amygdala

Estimate	t	p
0.25	7.68	2×10^{-14}
-5.15	-0.70	0.48
-0.48	-9.91	0
5.67	0.52	0.60

vmPFC

Name	Estimate	t	p
**Intercept**	0.18	2.20	0.028
**PAC**	24.51	1.18	0.24
**Load1**	-0.34	-2.84	0.004
**PAC : Load1**	-51.40	-1.67	0.10

Table S2. Mixed-effects GLM results for trial-by-trial correlations between RT and PAC. Related to Figure 2. Load 1 are tested against load 3 trials. No corrections for multiple comparisons.

PAC ~ 1 + $SC_{catCell}$ + load + $SC_{catCell}$ * load + (1 | patientID) + (1 | patientID:neuron-channelID)

Hippocampus

Name	Estimate	t	p
**Intercept**	-0.044	-3.14	0.002
**SC**	0.002	2.40	0.017
**Load1**	0.076	3.85	0.0001
**SC : Load1**	-0.004	-2.57	0.010

Amygdala

Estimate	t	p
0.003	0.33	0.74
-0.001	-0.75	0.45
-0.010	-0.76	0.45
0.002	1.48	0.14

Table S3. Mixed-effects GLM results for trial-by-trial correlations between PAC and spike counts of category neurons. Tested across all neuron-to-channel combinations involving significant PAC channels. Related to Figure 3. Load 1 are tested against load 3 trials. No corrections for multiple comparisons. SC = spike count.

$$PAC \sim 1 + SC_{pacCell} + load + SC_{pacCell} * load + (1 | patientID) + (1 | patientID:neuronID)$$

	Hippocampus				Amygdala		
Name	Estimate	t	p		Estimate	t	p
Intercept	-0.0565	-2.76	0.005		0.021	1.37	0.17
SC	0.0032	2.19	0.028		-3×10^{-5}	-0.02	0.98
Load1	0.0931	3.26	0.001		-0.054	-2.55	0.01
SC : Load1	-0.0043	-2.21	0.027		0.001	0.89	0.37

Table S4. Mixed-effects GLM results for trial-by-trial correlations between PAC and spike counts of PAC neurons. Related to Figure 4. Load 1 are tested against load 3 trials. No corrections for multiple comparisons. SC = spike count.

Session	Gender	Age	Seizure onset zone	Hippo	Amy	preSMA	dACC	vmPFC
P55cs	f	43	Right mesial temporal	1/14	21/14	12/14	10/14	2/14
P55cs_2	-	-	-	8/13	18/14	11/14	1/14	3/14
P56cs	m	48	Bilateral mesial temporal + orbitofrontal	4/12	21/13	5/6	4/14	8/7
P58cs	f	32	Right frontal neocortical	1/14	12/14	20/14	12/14	8/13
P60cs	m	67	Left mesial temporal	8/14	13/14	11/14	13/13	6/14
P61cs	f	52	Left mesial temporal	6/7	6/14	13/14	9/14	5/14
P61cs_2	-	-	-	2/7	8/14	18/12	0/7	8/13
P62cs	f	25	Left mesial temporal	0/0	12/7	7/7	0/6	5/7
P64cs	f	63	Right lateral temporal neocortical	0/7	17/6	25/14	47/12	23/13
P65cs	f	55	Bilateral independent temporal	5/0	18/7	2/14	1/14	7/7
P67cs	f	38	Bilateral mesial temporal	0/14	12/10	1/14	0/7	8/14
P68cs	m	54	Bilateral mesial temporal	16/0	7/3	7/14	2/13	4/14
P69cs	f	41	Not localized	8/15	7/14	0/7	10/14	11/15
P70cs	f	30	Right temporal	1/7	14/7	9/13	0/14	0/14
P70cs_2	-	-	-	1/7	5/6	5/14	0/14	1/12
P71cs	m	40	Not localized	0/14	3/14	3/14	10/14	11/14
P72cs	f	25	Not localized, bilateral independent	4/0	0/7	3/14	5/14	1/14
P73cs	f	58	Left mesial temporal	5/14	17/15	4/14	6/14	9/14
P76cs	f	24	Not localized	6/14	24/15	7/14	16/14	10/7
P77cs	f	46	Right auditory cortex	4/14	41/15	9/14	0/0	26/14
P78cs	f	54	Right anterior temporal	13/7	14/7	0/0	0/0	0/0

**P79cs**	f	42	Right anterior lateral temporal neocortex	17/7	28/8	20/14	12/14	18/16
**P79cs_2**	-	-	-	13/7	19/8	12/14	16/14	12/15
**P88T**	m	26	Right mesial temporal	22/0	6/0	0/0	0/0	0/0
**P89T**	f	45	Right frontal	22/28	18/14	0/0	0/14	0/0
**P90T**	m	20	Occipital cortex	13/17	29/7	0/0	0/0	0/0
**P90T_2**	-	-	-	11/27	2/11	0/0	0/0	0/0
**P90T_3**	-	-	-	13/27	0/11	0/0	0/0	0/0
**P91T**	f	59	Left cingulate cortex + insula + orbitofrontal	5/13	1/6	0/0	0/7	12/7
**P93T**	m	23	Left mesial temporal	4/7	3/0	0/0	0/7	0/0
**P96T**	f	58	Bilateral mesial temporal	6/0	5/14	0/0	0/0	0/0
**P101T**	f	25	Left neocortical temporal	16/28	5/8	0/0	0/0	0/0
**P101T_2**	-	-	-	8/27	6/8	0/0	0/0	0/0
**P103T**	m	49	Right mesial temporal	8/14	4/6	0/0	0/2	4/7
**P106T**	m	26	Multifocal	7/21	14/7	0/0	0/0	0/0
**P109T**	m	28	Multifocal	15/28	7/14	0/0	5/13	4/7
**P110T**	m	38	Right fusiform cortex	6/19	10/14	0/0	0/0	0/0
**P113T**	m	36	Right mesial temporal	13/28	19/14	0/0	0/0	0/0
**P113T_2**	-	-	-	11/21	7/14	0/0	0/0	0/0
**P116T**	m	28	Left amygdala	9/13	18/13	0/0	9/14	0/7
**P129T**	f	25	Bilateral mesial temporal	25/28	5/14	0/0	0/0	0/0
**P1802jh**	m	62	Right mesial temporal	12/16	0/0	0/0	0/0	0/0
**P1809jh**	m	45	Left inferior + middle frontal gyrus	1/8	0/0	0/0	0/0	0/0
**P1811jh**	f	27	Bilateral mesial temporal	10/8	0/0	0/0	0/0	0/0

Table S5. Patient demographics and neuron/channel count per area. For each area, the first number represents the neuron count, the second the number of clean micro LFP channels.

Supplementary Discussion

Role of high gamma band in PAC and WM

The high gamma band has been suggested to reflect processing of encoded information within the hippocampus[24,25,88]. In favor of this hypothesis, PAC in our study differed as a function of WM load only for frequencies involving the high gamma range. In line with earlier reports[82], our results show that storing more information in WM leads to lower estimates of theta-high gamma PAC because high gamma power is more broadly distributed across the theta cycle. A related finding is that in rats, the number of gamma cycles increases specifically in the high gamma range when the length of a running track increased[89,90], potentially signaling an increase in WM load. Moreover, in our study persistently active category neurons were more strongly phase locked to signals in the high gamma range when their preferred stimulus was maintained. No effects were observed for frequencies involving the low gamma band (30-55 Hz). Our study provides evidence for a specific role of the high gamma band in the processing and maintenance of WM content in the hippocampus, and reports load-dependent effects of theta-gamma PAC during WM maintenance.

In line with earlier studies[91–93], activity in the high gamma (70-140 Hz) frequency range was reflective of processing and maintenance in WM of encoded sensory information. Yamamoto and colleagues[91] found that in mice, the activity of high gamma oscillations (65-140 Hz) in the hippocampal-entorhinal system was related to successful WM maintenance. Synchronization in the high gamma band between the entorhinal cortex and hippocampus was stronger in correct than incorrect trials, and appeared shortly before a reversal of a decision when the animal initially made a wrong choice. The authors suggested that high gamma activity thus contributes to the explicit awareness of WM content. They did not find a relation of WM processes to activity in the lower gamma band (25-50 Hz). Similarly, Tort et al.[93] reported that PAC between theta and high gamma oscillations in the rat hippocampus was especially strong in time periods after a sensory cue has been represented, presumably involving processes of WM maintenance and decision making. They also did not observe PAC between theta and low gamma.

Relationship to findings in non-human primates.

In non-human primates[27,94–97], spiking of frontal cortex neurons is most informative about WM content during brief bursts of gamma oscillations, which occur when beta activity is low. Our

finding that the activity of content-tuned category neurons is related to gamma power and phase when their preferred stimulus was maintained in memory shows that a similar relationship is also present in the human hippocampus. In line with the NHP findings, interactions between WM content-related spiking and gamma rhythms in our study were especially strong when gamma power was high (**Extended Data Fig. 5d**). Moreover, gamma in our study was modulated by an underlying theta rhythm, showing that gamma activity was not monotonically sustained throughout the delay period. In contrast to the NHP findings, however, we did not observe information-carrying neurons that remained active during the maintenance period in frontal cortex. Indeed, no such neurons have been shown so far in human frontal cortex. A second notable difference is that in the hippocampus, low frequency modulations were related to the theta rather than the beta band as reported in NHP frontal cortex. It remains an open question of whether this difference in findings is due to a species difference, extent of training that the NHPs receive[98], or exact location of recordings within the frontal cortex.

Specificity of load-modulation of PAC to hippocampus

Although we observed significant theta-gamma PAC also in the amygdala and the vmPFC, PAC in those areas was not related to WM processes because it differed neither as a function of WM load nor was it related to WM-based behavior. Our findings are compatible with those of Johnson et al.[43], who also observed within-area theta-gamma PAC in orbitofrontal cortex (here vmPFC) that was not modulated by the WM task. In line with our results, within-region PAC changed as a function of WM task modulations within the MTL but not vmPFC. Earlier studies reported modulations in theta-gamma PAC in frontal cortex in the context of long-term memory processing rather than WM as we do here[20,69]. An important open question is whether our findings extend to dorsolateral PFC[99], which we did not examine here. Further, unlike in the hippocampus, category cells in the amygdala were not more strongly coupled to gamma when their preferred stimulus was maintained in WM. Earlier reports indicated that the amygdala plays a role in the maintenance of information in WM[12,100]. Our observation of stimulus-specific persistent activity of category neurons in the amygdala provides further evidence for this claim. However, our results indicate that the amygdala supports WM through a different mechanism than the hippocampus. Whether PAC and high gamma in the amygdala and medial frontal cortex serve a different role than that of the hippocampus during WM maintenance remains an open question.

Potential mechanisms

Top-down interactions between vmPFC and hippocampus did not directly involve persistently active category neurons, whose firing rate was informative about the currently maintained memoranda. Instead, we found that the activity of hippocampal PAC neurons, whose activity was not directly related to WM content maintenance per se, was coordinated with LFPs in the vmPFC. This suggests that instead of directly exerting cognitive control over WM-content processing neurons, vmPFC supported WM maintenance indirectly through an independent population of cells (i.e., PAC cells). These cells, in turn, locally interacted with WM content tuned cells to enhance WM fidelity. One way by which cognitive control is exerted is thought to be via monosynaptic projections from PFC to inhibitory interneurons in the hippocampus[41]. Malik et al.[41] observed that more top-down control led to enhanced signal to noise ratios of object-related spatial encoding and, at the same time, reduced overall network activity and inhibited feedforward processing in the hippocampus. Relatedly, we observed cognitive control related signals between hippocampal PAC neurons and vmPFC specifically for narrow-spiking neurons (see **Extended Data Fig. 8d,e**), which are thought to likely reflect inhibitory interneurons[86,87]. This therefore suggests the new specific hypothesis that the hippocampal PAC neurons we described are inhibitory interneurons that receive monosynaptic projections from PFC to coordinate local interactions with tuned, memoranda-maintaining neurons. Such interactions were here observed at the level of noise correlated firing rates as well as joint connectivity of both cell populations to local gamma oscillations. Further supporting this hypothesis is our finding that category neurons reduce their firing rate in load 3 compared to load 1 trials. It is possible that this is due to increased inhibition exerted as a function of increased need for cognitive control.

MEG studies of WM maintenance indicate that in the human temporal lobe, local PAC co-exists together with long-range theta phase synchronization to the frontal lobe[31,32]. These interactions were taken as evidence for an interplay between cognitive control and local WM content-specific processing. However, these non-invasive studies leave the mechanism by which these interactions could occur unclear – and, in particular, whether PAC at the M-/EEG level has functional consequences at the single cell level. Here, we now provide direct evidence that PAC neurons are related to both local processing and long-range interactions, thereby bridging these two levels of processing so commonly seen at the macroscopic level. We show that the ongoing WM content-specific processes of WM maintenance by memoranda-selective persistently active category neurons is accompanied by phase coupling to local gamma rhythms in the hippocampus. Gamma activity, in turn, was coordinated by the phase of theta activity. Crucially, single neurons

that followed the local interactions between theta phase and gamma amplitude played a functional role in receiving cognitive control signals from vmPFC, reflected by stronger cross-regional theta phase coupling in trials with higher WM load and faster RT. This effect was specific to PAC neurons. These observation are also in concordance with a model suggested by Mizuseki et al.[101], in which theta oscillations from vmPFC (instead of entorhinal cortex in their study) might act on a subset of hippocampal neurons (i.e., PAC neurons) to enable the interaction of local, within-hippocampal circuits (i.e., among category neurons) and support the maintenance of WM content through self-sustained activity (i.e., persistent activity). Together with our noise correlation results (see below), we thus suggest that PAC neurons facilitate the temporal coordination of hippocampal processes of WM maintenance with frontal cognitive control processes.

Related work on role of theta and vmPFC in long-range interactions

Earlier studies reported functional differences of PAC involving slow (2-5 Hz) and fast theta frequencies (5-9 Hz)[20,21]. To examine whether we see similar differences, we also compared PAC between the load conditions separately for high and low theta. PAC differed significantly only in the hippocampus and only for high gamma power combined with either low or high theta frequency bands, with the strongest differences in the fast theta band (see **supplementary Table S1**).

Liebe and colleagues[102] observed enhanced cross-regional phase coupling in the theta range between single neurons in macaque V4 and LFPs recorded in lateral prefrontal cortex during a WM maintenance period. While this prior study shows that phase locking of V4 neurons to frontal theta activity was stronger in successful as compared to error trials, this study left it unclear whether such phase coupling was related to the cognitive control of WM content. Also, in this prior study, theta coupling was not related to modulations of WM load nor to interactions with local maintenance processes of WM content in higher frequencies.

We note that while vmPFC is known to be involved in top-down control processes[103–105], especially in interaction with the hippocampus[106–108], we are the first to show that it is engaged in the long-range cognitive control of the maintenance of WM information in the hippocampus.

Further discussion on noise correlations

In addition, we here show that noise correlations among PAC and category neuron pairs predicted WM-related behavior in a way that other pairs of neurons did not, thereby showing their behavioral relevance. Noise correlations between PAC and category neurons were stronger in fast than slow RT trials, and this effect was significantly stronger than for randomly selected pairs of non-PAC and category neurons. Our report describes a functional role for noise correlations between neurons in humans. This is in contrast to earlier work in macaques, which showed increased decodability of WM content but did not provide a link with WM-dependent behavior[36]. We conclude that cognitive control exerted through PAC neurons can stabilize WM representations and thereby enhance the readout of WM content, leading to faster RTs. This finding suggests that noise correlations among PAC and memoranda-selective persistently active neurons might be a mechanism for stabilizing WM representations and their underlying persistent neural activity against noise or distractors for long timescales. In line with this interpretation, noise correlations in our study were especially beneficial to behavior in load 3 where competing WM representations co-exist in the neural population. A hypothesis from our work that remains for further exploration is that noise correlations become stronger in the presence of distractors to enhance control over neural activity.

Supplementary References

88. Colgin, L. L. Rhythms of the hippocampal network. *Nat Rev Neurosci* **17**, 239–249 (2016).

89. Gupta, A. S., Meer, M. A. A. V. D., Touretzky, D. S. & Redish, A. D. Segmentation of spatial experience by hippocampal theta sequences. *Nat Neurosci* **15**, 1032--1039 (2012).

90. Zheng, C., Bieri, K. W., Hsiao, Y. T. & Colgin, L. L. Spatial Sequence Coding Differs during Slow and Fast Gamma Rhythms in the Hippocampus. *Neuron* **89**, 398--408 (2016).

91. Yamamoto, J., Suh, J., Takeuchi, D. & Tonegawa, S. Successful execution of working memory linked to synchronized high-frequency gamma oscillations. *Cell* **157**, 845--857 (2014).

92. Cabral, H. O. *et al.* Oscillatory Dynamics and Place Field Maps Reflect Hippocampal Ensemble Processing of Sequence and Place Memory under NMDA Receptor Control. *Neuron* **81**, 402–415 (2014).

93. Tort, A. B. L. *et al.* Dynamic cross-frequency couplings of local field potential oscillations in rat striatum and hippocampus during performance of a T-maze task. *Proc National Acad Sci* **105**, 20517--20522 (2008).

94. Lundqvist, M. *et al.* Gamma and Beta Bursts Underlie Working Memory. *Neuron* **90**, 152–164 (2016).

95. Lundqvist, M., Herman, P., Warden, M. R., Brincat, S. L. & Miller, E. K. Gamma and beta bursts during working memory readout suggest roles in its volitional control. *Nat Commun* **9**, 394 (2018).

96. Bastos, A. M., Loonis, R., Kornblith, S., Lundqvist, M. & Miller, E. K. Laminar recordings in frontal cortex suggest distinct layers for maintenance and control of working memory. *Proc National Acad Sci* **115**, 1117–1122 (2018).

97. Lundqvist, M. *et al.* Working memory control dynamics follow principles of spatial computing. *Nat Commun* **14**, 1429 (2023).

98. Miller, J. A., Tambini, A., Kiyonaga, A. & D'Esposito, M. Long-term learning transforms prefrontal cortex representations during working memory. *Neuron* **110**, 3805-3819.e6 (2022).

99. Owen, A. M. The Functional Organization of Working Memory Processes Within Human Lateral Frontal Cortex: The Contribution of Functional Neuroimaging. *Eur. J. Neurosci.* **9**, 1329–1339 (1997).

100. Schaefer, A. & Gray, J. R. A Role for the Human Amygdala in Higher Cognition. *Rev Neuroscience* **18**, 355–364 (2007).

101. Mizuseki, K., Sirota, A., Pastalkova, E. & Buzsáki, G. Theta Oscillations Provide Temporal Windows for Local Circuit Computation in the Entorhinal-Hippocampal Loop. *Neuron* **64**, 267–280 (2009).

102. Liebe, S., Hoerzer, G. M., Logothetis, N. K. & Rainer, G. Theta coupling between V4 and prefrontal cortex predicts visual short-term memory performance. *Nat Neurosci* **15**, 456–462 (2012).

103. Chai, W. J., Hamid, A. I. A. & Abdullah, J. M. Working Memory From the Psychological and Neurosciences Perspectives: A Review. *Front Psychol* **9**, 401 (2018).

104. Yin, S., Bi, T., Chen, A. & Egner, T. Ventromedial Prefrontal Cortex Drives the Prioritization of Self-Associated Stimuli in Working Memory. *J Neurosci* **41**, 2012–2023 (2021).

105. Badre, D. & Nee, D. E. Frontal Cortex and the Hierarchical Control of Behavior. *Trends Cogn Sci* **22**, 170–188 (2018).

106. Jin, J. & Maren, S. Prefrontal-Hippocampal Interactions in Memory and Emotion. *Frontiers Syst Neurosci* **9**, 170 (2015).

107. Gluth, S., Sommer, T., Rieskamp, J. & Büchel, C. Effective Connectivity between Hippocampus and Ventromedial Prefrontal Cortex Controls Preferential Choices from Memory. *Neuron* **86**, 1078–1090 (2015).

108. Günseli, E. & Aly, M. Preparation for upcoming attentional states in the hippocampus and medial prefrontal cortex. *Elife* **9**, e53191 (2020).
