# Supplementary material for: Control of working memory by phase–amplitude coupling of human hippocampal neurons
Source: Nature. 2024 Apr 17;629(8011):393–401. doi: 10.1038/s41586-024-07309-z (PMC11078732; doi:10.1038/s41586-024-07309-z)
Supplement: Supplementary file 2 — Reporting Summary [file 41586_2024_7309_MOESM2_ESM.pdf]

nature portfolio

Corresponding author(s): Ueli Rutishauser

Last updated by author(s): Feb 21, 2024

Reporting Summary

Nature Portfolio wishes to improve the reproducibility of the work that we publish. This form provides structure for consistency and transparency in reporting. For further information on Nature Portfolio policies, see our Editorial Policies and the Editorial Policy Checklist.

Statistics

For all statistical analyses, confirm that the following items are present in the figure legend, table legend, main text, or Methods section.

n/a	Confirmed	
☐	☒	The exact sample size (*n*) for each experimental group/condition, given as a discrete number and unit of measurement
☐	☒	A statement on whether measurements were taken from distinct samples or whether the same sample was measured repeatedly
☐	☒	The statistical test(s) used AND whether they are one- or two-sided<br>*Only common tests should be described solely by name; describe more complex techniques in the Methods section.*
☐	☒	A description of all covariates tested
☐	☒	A description of any assumptions or corrections, such as tests of normality and adjustment for multiple comparisons
☐	☒	A full description of the statistical parameters including central tendency (e.g. means) or other basic estimates (e.g. regression coefficient) AND variation (e.g. standard deviation) or associated estimates of uncertainty (e.g. confidence intervals)
☐	☒	For null hypothesis testing, the test statistic (e.g. F, t, r) with confidence intervals, effect sizes, degrees of freedom and P value noted<br>*Give P values as exact values whenever suitable.*
☒	☐	For Bayesian analysis, information on the choice of priors and Markov chain Monte Carlo settings
☒	☐	For hierarchical and complex designs, identification of the appropriate level for tests and full reporting of outcomes
☐	☒	Estimates of effect sizes (e.g. Cohen's *d*, Pearson's *r*), indicating how they were calculated

Our web collection on statistics for biologists contains articles on many of the points above.

Software and code

Policy information about availability of computer code

Data collection	Neurophysiological data were collected using the ATLAS (Neuralynx Inc. @ CSMC and UHN) or Blackrock system (Blackrock Neurotech Inc. @ JHU) and hybrid Behnke-Fried depth electrodes (Ad-Tech Inc.). See detailed description in the Methods section. Data is available for download at https://dandiarchive.org/dandiset/000673
Data analysis	Data analyses were performed using MATLAB 2019b, Fieldtrip 20200409, EEGLAB 2019.1, Python 3.10, and bycycle 1.1. Spike sorting was done using OSort 4.1 (https://rutishauserlab.org/osort). Anatomical data analysis was performed using FreeSurfer 6 (surfer.nmr.mgh.harvard.edu). Localization of electrodes was visualized using the CIT168 atlas (https://osf.io/r2hvk/) and the brainnetome atlas (https://atlas.brainnetome.org/). Bayes factors were calculated using the BayesFactor toolbox (https://zenodo.org/records/7006300). Specific functions used in this study are described in the Methods section. Code is available for download at https://zenodo.org/doi/10.5281/zenodo.10494533

For manuscripts utilizing custom algorithms or software that are central to the research but not yet described in published literature, software must be made available to editors and reviewers. We strongly encourage code deposition in a community repository (e.g. GitHub). See the Nature Portfolio guidelines for submitting code & software for further information.

Data

Policy information about availability of data

All manuscripts must include a data availability statement. This statement should provide the following information, where applicable:

- Accession codes, unique identifiers, or web links for publicly available datasets
- A description of any restrictions on data availability
- For clinical datasets or third party data, please ensure that the statement adheres to our policy

All data used in this study are publicly available in the DANDI Archive59 (https://dandiarchive.org/dandiset/000673/0.240118.2135). The published data set contains the timestamps and waveforms of the sorted neurons, LFPs, electrode coordinates, behavioral data, as well as the stimuli, triggers, experimental parameters, anonymized patient metadata of each session.

Research involving human participants, their data, or biological material

Policy information about studies with human participants or human data. See also policy information about sex, gender (identity/presentation), and sexual orientation and race, ethnicity and racism.

Reporting on sex and gender	The methods section contains the self-reported gender for all patients.
Reporting on race, ethnicity, or other socially relevant groupings	We do not use nor report socially relevant categorization variables in our study.
Population characteristics	We studied a group of 36 patients (21 females, 15 males) with an average age of 40.5 ± 13.8 years. All patients were diagnosed with pharmacologically-intractable epilepsy. Supplementary table S5 provides information about each patient.
Recruitment	Patients undergoing invasive electrophysiological recording for clinical purposes were recruited and consented to participate in this research study. Patients who were capable of and willing to participate in the task were recruited. All patients who spoke english and had a sufficient level of cognitive function were offered participation. Potential biases include subjects who do not speak english and low cognitive function.
Ethics oversight	The study was approved by the institutional review boards of Cedars-Sinai Medical Center, Toronto Western Hospital, and John's Hopkins School of Medicine. Patients provided informed consent.

Note that full information on the approval of the study protocol must also be provided in the manuscript.

Field-specific reporting

Please select the one below that is the best fit for your research. If you are not sure, read the appropriate sections before making your selection.

☒ Life sciences ☐ Behavioural & social sciences ☐ Ecological, evolutionary & environmental sciences

For a reference copy of the document with all sections, see nature.com/documents/nr-reporting-summary-flat.pdf

Life sciences study design

All studies must disclose on these points even when the disclosure is negative.

Sample size	Our analysis is based on 1452 neurons recorded from 36 patients. No statistical methods were used to pre-determine sample sizes.
Data exclusions	We excluded individual channels and trials that contained epileptic activity, electrical artifacts or movement-related electrical noise. The methods section contains detailed descriptions of the criteria used to exclude these data. These exclusion criteria were not pre-established but are commonly used.
Replication	The analyses were performed at the single neuron and channel level. The effects reported in the study were consistent and replicated across 36 subjects.
Randomization	Our design is a within-subject analysis: all the patients were in the same analysis set and had all types of trials. We performed permutation testing where appropriate to ensure statistical validity of our results.
Blinding	Patients were not aware of the goals of the study. There was no subjective measurement or decision that the investigator needed to make during the experiment. All the data are collected and analyzed off-line. Data collection and analysis were not performed blind to the conditions of the experiments as conditional information is required for further analyses.

Reporting for specific materials, systems and methods

We require information from authors about some types of materials, experimental systems and methods used in many studies. Here, indicate whether each material, system or method listed is relevant to your study. If you are not sure if a list item applies to your research, read the appropriate section before selecting a response.

Materials & experimental systems

n/a	Involved in the study
☒	☐ Antibodies
☒	☐ Eukaryotic cell lines
☒	☐ Palaeontology and archaeology
☒	☐ Animals and other organisms
☒	☐ Clinical data
☒	☐ Dual use research of concern
☒	☐ Plants

Methods

n/a	Involved in the study
☒	☐ ChIP-seq
☒	☐ Flow cytometry
☒	☐ MRI-based neuroimaging
